# Individual and Environmental Factors Associated with Proteinuria in Korean Children: A Multilevel Analysis

**DOI:** 10.3390/ijerph16183317

**Published:** 2019-09-09

**Authors:** Suhee Kim, Ju-Yeon Uhm

**Affiliations:** 1 School of Nursing and Research Institute of Nursing Science, Hallym University, Chuncheon-si, Gangwon-do 24252, Korea; 2 Department of Nursing, Pukyong National University, Busan 48513, Korea

**Keywords:** proteinuria, body mass index, environmental pollution, urbanization

## Abstract

Proteinuria is a significant sign of childhood renal disorders. However, little is known about how sociodemographic and environmental factors are related to the presence of proteinuria among children and adolescents. This paper focuses on the prevalence of proteinuria and its risk factors among children and adolescents. This study conducted a secondary analysis of data from the 2016 Sample Schools Raw Data of Health Examination for School Students (SSRDHESS). Data collected from 27,081 students who had undergone a health screening were analyzed using Chi-square tests, independent *t*-tests, and multilevel logistic regression analysis. The prevalence of proteinuria was higher in the thin group than in the normal weight group (adjusted odds ratio (aOR) = 1.77; 95% confidence interval (CI) = 1.34–2.33) and lower in the overweight/obese group (aOR = 0.64; 95% CI = 0.51–0.80). Additionally, those in metropolitan and small–medium sized cities had a proteinuria prevalence about 1.5-fold higher than that of those in rural areas (95% CI = 1.08–2.02, 95% CI = 1.19–1.92, respectively). Proteinuria was associated with environmental pollution, including smoking rate, ambient particulate matter and heavy metals in drinking water (aOR = 1.10; 95% CI = 1.01–1.20; aOR = 1.06; 95% CI = 1.01–1.11, aOR = 1.001; 95% CI = 1.0001–1.0015). These results suggest that to improve health management effectiveness, kidney disease prevention efforts for children and adolescents should focus on geographical area and environmental pollution, as well as body weight as individual factors.

## 1. Introduction 

In children, proteinuria may involve only transient findings, usually accompanying a non-specific viral infection. However, proteinuria, including albuminuria, is a significant sign of childhood renal disorders [1].

The prevalence of proteinuria can be as high as 6.2% in routine urine testing among school-aged children [2,3,4] and albuminuria in children is estimated to be about 5.7–7.3% in boys and 12.7–15.1% in girls [5]. According to previous studies, the prevalence of proteinuria is associated with an adolescent’s age, sibling diabetes [3], more senior grades in school [6], Tanner staging [7], and hypertension [3,8]. Compared with normal weights, obesity has been shown to be associated with a higher risk of kidney disease [9], and proteinuria was also found to be higher in obese individuals [10]. In a pediatric study, the prevalence of proteinuria was higher in obese than in non-obese children [8]. However, the higher prevalence of proteinuria was also found in thin groups as well as an obese group [11]. In addition, low body weight was a risk factor for proteinuria in a multiracial pediatric population [12]. In this way, evidence regarding the association of body mass index (BMI) with proteinuria incidences is ambiguous. Additionally, associations between individual factors such as duration of sleep and fast food consumption and prevalence of proteinuria remain unknown. 

Several environmental factors including industrial growth, environmental pollution, and the use of food containers and chemical products have been posited as probable causes for the prevalence of proteinuria. Several studies have demonstrated evidence regarding the relationship between exposure to environmental factors such as bisphenol A (BPA), heavy metals (including lead, cadmium, and chromium), and cigarette smoking and direct kidney damage causing albuminuria in the pediatric population [13]. Additionally, exposure to ambient fine particulate matter with an aerodynamic diameter ≤2.5 μm (PM_2.5_) negatively affects renal function in adults [14]. Urbanization is also a risk factor for renal disorder, related to lifestyle habits such as induced sedentary lifestyle, high-calorie diets, and lack of sleep in adult populations [15]. However, associations between environmental factors such as geographic features, urbanization, PM_2.5_, ambient air particulate matter with aerodynamic diameters ≤10μm (PM_10_)_,_ and neighborhood food store environments and proteinuria have yet to be revealed in the pediatric population. In addition, there are differences in these environmental characteristics between regions and countries. Therefore, it is necessary to identify the environmental factors influencing youth people’s health while keeping the region in mind.

This study aimed to identify proteinuria’s prevalence and the relationship between undifferentiated or obscure factors from existing studies and proteinuria in children and adolescents. In addition, this study aimed to verify possible individual and regional factors influencing proteinuria among children and adolescents using the latest regional and national data. This study was guided by seven research questions: 

1. What is the prevalence of proteinuria in children and adolescents? 

2. What are the individual characteristics of the subjects (e.g., demographic characteristics, health status, health behaviors, and so on)? 

3. What are the regional characteristics (e.g., geographic characteristics and pollution, among others)? 

4. What are the correlations between regional characteristics? 

5. What are the differences in the prevalence of proteinuria according to individual characteristics? 

6. What are the differences in the prevalence of proteinuria according to regional characteristics? 

7. What factors ultimately affect proteinuria when both individual and regional characteristics are considered? 

## 2. Materials and Methods

### 2.1. Study Design and Participants

This was a secondary data analysis study to investigate the prevalence of proteinuria and identify risk factors influencing it. Individual- and regional-level data were merged. The individual-level data were obtained from the 2016 Sample Schools Raw Data of Health Examination for School Students (SSRDHESS) and regional-level data were obtained from the Statistics Office and the daily newspaper articles in Hankookilbo.

The Health Examination for School Students (HESS) is conducted for school-aged children and adolescents by the Korean Ministry of Education [16]. It is categorized into three domains: physical development examinations, health screenings, and health surveys. The physical development examination measures the height and weight of students; health screening includes a review of the human body, blood tests, and chest X-rays; and the health surveys collect data about sleep, exercise, and diet. Physical development examinations and health surveys are conducted annually for all students. Health screenings are conducted in hospitals only for specific grades: first, fourth, seventh, and tenth.

Though HESS is administered to all students throughout the country, the SSRDHESS, without personal information, is provided for study via an open website. The sample schools were first stratified by regions, city sizes, and three school types (elementary, middle, and high schools). The sample schools were then selected based on probability proportional to size sampling in each stratum, and one class was randomly selected per grade level. In other words, the Korean Ministry of Education selected 765 schools out of 11,554 schools nationwide; that is, the students selected for research (82,883) make up 1.42% of the total population of students nationwide (5,833,184). The Korean Ministry of Education analyzes these data to inform student health policies and provides such data to the public for research purposes.

The 2016 SSRDHESS included 82,883 students from 765 schools. Of these, only 27,671 students were eligible for the health screening. Thirty-three students were excluded because of diagnosed diseases, 93 proteinuria examinations were missing data, and 464 cases from Sejong City had data problems. This gave a total of 27,081 students living in 16 regions for the final analysis.

This study was exempted from review by the Institutional Review Board because of the public availability of the data.

### 2.2. Measures

Proteinuria: according to the Kidney Disease Improving Global Outcomes (KDIGO) guideline, an initial screening method for proteinuria is proposed in the following order, and in all cases, an early morning urine sample is preferred: (1) urine albumin-to-creatinine ratio, (2) urine protein-to-creatinine ratio, (3) reagent strip urinalysis for total protein with automated reading, and (4) reagent strip urinalysis for total protein with manual reading [17]. This study used the following definition using a reagent strip. A positive result of the urine dipstick test (1+ more) was taken as indicating the presence of proteinuria. Negative findings, weak positive findings, or equivocal data (±) were considered normal [16]. Because this study was a secondary data analysis study, we could not confirm whether the urine stick was read automatically or manually or whether the urine stick was used first thing in the morning. In addition, the method proposed by KDIGO and the method defined in this study are, strictly speaking, closer to albuminuria, but we have used the term proteinuria in this study because albumin is one part of urinary protein.

Individual-level variables based on known independent factors affecting proteinuria in previous research included demographic characteristics (sex and grade), health status (body mass index (BMI) and blood pressure), and health behaviors (exercise, duration of sleep, and fast food intake). BMI cutoffs for classifying thinness, normal weight, overweight, or obesity were based on the criteria of the International Obesity Task Force (IOTF) [18]. Exercise was divided into two cases (none vs. three or more days a week) and fast food intake was classified by consumption of hamburgers, pizza, and fried food more than once a week [16]. 

Regional-level variables included geographic characteristics (middle-south, east-west, and urbanization) and data related to pollution including smoking rate, PM_10_, PM_2.5_, the number of heavy metals in drinking water, and the number of convenience stores per square km. South Korea is surrounded by the sea in the east, west, and south, with high mountains in the east. The eastern portion is characterized by high mountains and narrow coastal plains, and the western portion by wide fields and broad coastal plains. Korea can be divided into the northern region, the middle region, and the southern region. North Korea is often called the northern region. In South Korea, the Sobaek mountain ranges and the downstream area of the Geum river can be divided into the middle region and the southern region [19]. The middle region includes Seoul City, Incheon City, Daejeon City, Sejong City, Gyeonggi-do, Chungcheongbuk-do, Chungcheongnam-do, and Gangwon-do. The southern region includes Gwangju, Busan, Daegu, Ulsan, Jeollabuk-do, Jeollanam-do, Gyeongsangbuk-do, Gyeongsangnam-do, and Jeju. In addition to these regions, the eastern and western portions were additionally divided. We classified Gangwon-do, Gyeongsangbuk-do, Gyeongsangnam-do, Daegu, Ulsan, and Busan as part of the eastern region and the remaining cities as part of the western region. The urbanization variables were provided by SSRDHESS with classified data in three categories: metropolitan, small–medium sized city, and rural area. Variables related to air pollution such as smoking rate, PM_10_, and PM_2.5_ were collected from the data of the Statistical Office in 2016 [20,21,22]. As an indicator of water pollution, the number of positive results of tests to identify whether there were heavy metals in drinking water during the last five years (January 2012–July 2017) were used [23]. The data on heavy metals in drinking water were collected from newspaper articles that have been reported over the past five years; such reports were made when heavy metals in drinking water such as tap water, ground water, and mineral springs exceeded standard levels. Convenience stores are small retailers that sell a variety of products, including groceries, snacks, soft drinks, and tobacco; students in particular visit convenience stores for snacks, instant noodles, and microwave convenience foods. Hence, we included the number of convenience stores per square km as an indicator of accessibility to environmental hormones [24].

### 2.3. Statistical Analyses

We used descriptive statistics to identify proteinuria’s prevalence and individual and regional characteristics, and the associations among regional variables were analyzed using Pearson’s correlations. Chi-square tests and independent *t*-tests were performed to assess the associations between the individual and regional factors and proteinuria in children and adolescents using SPSS 21.0 (IBM Corp., Armonk, NY, USA).

Multilevel analysis was performed to analyze not only the individual factors influencing proteinuria in children and adolescents, but also the regional differences. Multilevel logistic regression analysis identified the effects of individual- and regional-level factors on the proteinuria in students using “melogit” commands in the STATA/IC 14.0 software package. Individual characteristics were first-level and regional characteristics were second-level independent variables. The dependent variable was the result of the urine protein test (1 = positive reaction, 0 = normal/negative reaction). 

Initially, a null model without any independent variables was established. This model was used to determine how the two different levels contributed to variations in student proteinuria status. Following the assessment of the significance of the variation at each level, conditional models were estimated in Models 1 and 2. In Model 1, students’ individual-level variables were included as predictors in a series of multilevel logistic regression models. In Model 2, students’ individual- and regional-level variables were included. 

## 3. Results 

Of the 27,081 participants, 529 students (2.0%) tested positive for proteinuria. Males made up 51.6% of the sample. They were distributed between first grade (20.2%), fourth grade (20.1%), seventh grade (26.8%), and tenth grade (32.9%). On the basis of the IOTF criteria, 6.7% of subjects were thin, 65.5% were normal weight, and 27.8% were overweight or obese. The means of systolic blood pressure and diastolic blood pressure were 102.55 (±12.28) mmHg and 61.69 (±8.16) mmHg, respectively. Among participants, 60.7% exercise less than three days a week, 70.3% averaged less than 8 h of sleep per night, and 70.6% ate fast food such as hamburgers more than once a week (Table 1).

In the 16 regions, the mean smoking rate was 22.28% (±1.69%); the means of PM_10_ and PM_2.5_ were 44.88 (±4.30) μg/m³ and 25.19 (±2.59) μg/m³, respectively. The mean number of positive results of tests to detect heavy metals in drinking water was 146.81 (±168.27). The number of convenience stores per square km in the region ranged from 0.34 to 12.47, with an average of 2.12 (±2.92). The regional characteristics showed a significant correlation with each other. Smoking rate was negatively associated with PM_10_ (r = −0.11, *p* < 0.001), PM_2.5_ (r = −0.18, *p* < 0.001), and the number of convenience stores per square km (r = −0.46, *p* < 0.001). PM_10_ was positively associated with PM_2.5_ (r = 0.75, *p* < 0.001), heavy metals in drinking water (r = 0.03, *p* < 0.001), and the number of convenience stores per square km (r = 0.27, *p* < 0.001) (Table 2).

Among the 27,081 participants, female students showed a higher prevalence than male students (2.2% vs. 1.7%, respectively; *p* = 0.003). Proteinuria also showed differences by grade (*p* < 0.001), BMI (*p* < 0.001), duration of sleep (*p* < 0.001), and fast food intake (*p* = 0.012) (Table 1). 

In terms of regional characteristics, while 1.4% of those in rural areas had positive reactions for proteinuria, the figure was 2.3% for those in metropolitan areas (*p* < 0.001). Moreover, proteinuria also showed differences in the middle-south area (*p* = 0.001), east-west area (*p* < 0.001), and according to the prevalence of convenience stores (*p* = 0.017) (Table 3). 

The variables that showed differences in the univariate analysis were included for the multilevel analysis. In addition, we included smoking rate, PM_10_, PM_2.5_, and the number of heavy metals in drinking water as environmental pollution indicators, which we aimed to investigate in this study. The variance inflation factor values for the correlation between independent variables were 1.014 to 5.469, less than 10, confirming that there was no problem with multicollinearity.

For random-effect parameters, in the null model, the likelihood-ratio test indicated that the proportion of variations in proteinuria varied by region (*p* < 0.001). Model 1 showed regional variances even after adjusting for individual-level factors (*p* < 0.001). Finally, in Model 2, after adjusting for individual and regional factors, the variances in the regional level were nearly 0, indicating that both level variables were sufficient to explain proteinuria in students. 

Among the fixed effects in Model 2, seventh-grade students had a proteinuria prevalence 5.75 times higher than that of first-grade students (95% CI = 3.84–8.63) and thin students had positive reactions for proteinuria 1.77 times higher than normal-weight students (95% CI = 1.34–2.33) (Table 4).

In terms of regional characteristics, those in metropolitan areas and small–medium sized cities had a proteinuria prevalence about 1.5-fold higher than those in rural areas (95% CI = 1.08–2.02, 95% CI = 1.19–1.92, respectively). As variables related to environmental pollution, smoking rate, PM_10_, heavy metals in drinking water, and the number of convenience stores slightly increased proteinuria’s prevalence among students (aOR = 1.10, 95% CI = 1.01–1.20; aOR = 1.06, 95% CI = 1.01–1.11; aOR = 1.001, 95% CI = 1.0001–1.0015; aOR = 1.09, 95% CI = 1.05–1.13, respectively).

## 4. Discussion

In this study, the prevalence of proteinuria was 2.0%. This is lower than the 5.7% in Taiwanese children [6], but shows a higher prevalence than the 0.2% found among Korean children from 1998 to 2004 [2]. The finding is similar to the SSRDHESS results from 2012 and 2015, of 1.6% to 1.9% respectively [16], but the trend is increasing slightly as a result of changes in lifestyle and environment, such as increased consumption of fast food, decreased amount of sleep, and increased environment pollution.

Our results demonstrated that a low BMI is a risk factor for proteinuria. Previous studies have debated the association between proteinuria and BMI, with some reporting that high BMI is associated with proteinuria [8,25], and others supporting our findings that the prevalence of proteinuria was higher in the lower BMI group than in normal or higher BMI groups [3,26]. Mechanisms concerning the association between thinness and proteinuria remain obscure and only a few hypotheses exist. Transient or orthostatic proteinuria in children are very common. This finding can be related to increased activities in the low BMI group, in contrast with the low levels of physical activity in the obese group. One possible mechanism is that body fat content modulates proteinuria, which was explained with the hypothesis that the space between the aorta and the superior mesenteric artery is modulated by BMI [27]. In other words, proteinuria is associated with renal nutcracker syndrome [28], which might be related to thinness. However, in a recent study, neither proteinuria nor BMI were correlated with the ratio of the peak velocity and diameters of the left renal vein [29]. Another mechanism is the association between reduced muscle mass in those with low BMI and inflammation [30], inflammation could induce microalbuminuria [31].

In this study, the prevalence of proteinuria was higher among girls than boys. One study reported that proteinuria was more frequent in boys than girls [26], which has been explained by a higher incidence of orthostatic proteinuria in boys [32]. However, another study, like this study, reported that the prevalence of proteinuria was higher in girls than in boys [33]. Therefore, further study is needed to investigate covariates to explain the differences in prevalence between girls and boys. 

Interestingly, the incidence of proteinuria was associated with a duration of sleep of less than 8 h as an individual factor. This result is similar to a finding in a population with young children in Japan [34]. The mechanisms of lack of sleep to cause proteinuria are still unrevealed. Considering BMI in this study, lack of sleep is unlikely to act as a significant intermediate variable in determining associations between low BMI and proteinuria. The reason is because there is a growing consensus on associations between shorter sleep duration and higher BMI in children and adolescents (OR 2.15; 95% CI, 1.64–2.81) [35,36]. Chen et al. (2017) suggested that the possibility of higher incidence in populations with shorter durations of sleep could be related to systematic inflammation. The relative risk of proteinuria in shorter sleepers was 1.47 in an adult population (95% CI, 1.26–1.72) [37]. Short sleep is associated with various health problem such as hypertension [38] and metabolic syndrome [39] in adult populations. Furthermore, shorter sleep could be related to more physical activities during wake time as the associations of transient proteinuria with posture and strenuous exercise or febrile illness are well established and orthostatic proteinuria occurs commonly in adolescents [40]. There is a lack of research on risk factors for lack of sleep in children with proteinuria; further studies are needed. 

Additionally, there was a link between fast food consumption and proteinuria, although the underlying mechanism for this association remains unknown. Poor diet quality was found to be a significant factor of microalbuminuria in the young adult population [41]. Prior reports pointed out that fast-food meals are related to inflammation and oxidative stress [42] as well as diabetes, metabolic problem, and chronic illness [43].

In our multivariate analysis, the fourth, tenth, and seventh grades were risk factors for proteinuria. Chen et al. demonstrated that both seventh-grade boys and girls had a higher odds ratio of proteinuria than first-grade students [6]. There is evidence that urinary albumin excretion is estimated to be the lowest in children younger than six years, followed by an increase through adolescence with a peak at age 15–16 [44]. In addition to transient proteinuria, another possible explanation for grade being a factor is exposure to BPA and phthalates added to several categories of food, canned soup, and plastic containers for food and beverages [45,46]. Children in the high-exposure group for phthalates and BPA have increased risk of microalbuminuria [47,48]. These studies have relevance to the positive association between prevalence of proteinuria and the number of convenience stores in this study. While there is no study examining the number of convenience stores and proteinuria, there are several studies regarding the association of the food store environment and BMI at peri puberty. Prior studies are inconsistent with our findings regarding the link between lower BMI and proteinuria. Living farther from fast food and full-service restaurants has been found to be associated with lower BMI [49]. The authors pointed out the higher tendency of higher-calorie foods found in fast food or full-service restaurants. The presence of convenience, small-sized grocery stores and limited-service restaurants in neighborhoods was associated with a higher BMI in children [50,51]. Therefore, it can be postulated that proteinuria could be explained with environmental factors such as convenience stores rather than direct effects of BMI. Additionally, BPA levels have increased and the consumption of instant noodle cups and canned foods has increased the risks of BPA levels in the adult population [52]. Korea has been ranked the highest worldwide in per capita instant noodle consumption [53], with 74.6 servings per capita in 2018. The consumption of ultra-processed foods for ready-to-heat and ready-to-eat products has increased as a result of convenience and accessibility. Ultra-processed diets are associated with risks of various health problems in children and adolescents [54,55]. Polystyrene is widely used in food packing; its nanoparticles affect cell viability and induce apoptosis in cells [56]. 

Additionally, we found several geographic factors related to proteinuria’s prevalence in this study. First, proteinuria’s prevalence is higher in eastern than western regions, and the southern area showed a higher prevalence of proteinuria than the inland area. The associations between physical geography and proteinuria’s prevalence may be related to environmental pollution exposure. The southern and eastern areas of South Korea have major industrial complexes with severe air pollution [57,58,59,60]. Combustion and industry are the most common and influential sources of PM_10_ [61]. Mechanisms underlying the association of air pollution with renal function have been explained as inflammation and endothelial dysfunction by transition metals induced in reactive oxygen species [62,63]. Accordingly, oxidative stress and subsequent endocardial dysfunction affect the glomerular filtration rate (GFR) [64,65]. Moreover, the prevalence of proteinuria was associated with the level of PM_10_ in this study. Exposure to PM_10_ was associated with decreases in estimated glomerular filtration rate (eGFR) levels [66] and an elevated risk of microalbuminuria [67]. Additionally, in our analysis, PM_10_ was strongly correlated with PM_2.5_. Chronic PM_2.5_ exposure also negatively affected renal function decline [14]. Additionally, smoking was a risk factor for proteinuria. In adolescents, active smoking and secondhand smoke have been associated with decreased eGFR [68], and a relationship has been found between smoking and albuminuria in adult populations, as well as secondhand smoke exposure and proteinuria in children with chronic kidney disease [13].

Concerning this, we have found that residential areas are associated with proteinuria prevalence, with a higher incidence in the populations of metropolitan areas or medium-to-small cities than in rural regions. There are increased risks caused by urbanization and industrialization with a high standard of living, with urban or suburban areas having severe air pollution. Motor vehicle and secondary aerosol sources contribute to the production of particulate matter [61]. Urbanization is associated with reduced renal function [15], showing higher odds of reduced eGFR in more urbanized communities [69]. Furthermore, urbanized areas have more convenience stores, giving children more access to them. Children with chronic kidney disease consumed more sodium, protein, and calories, which is heavily driven by fast food consumption [70]. 

In this study, heavy metals in drinking water increased proteinuria’s prevalence. Exposure to heavy metals such as lead and cadmium [71], as well as to mercury [72], could be measured as renal or oxidative stress biomarkers. Indeed, environmental exposure to chromium and arsenic in children is associated with kidney injury molecule-1 [73] and albuminuria is associated with urinary cadmium and lead levels [74]. In 2014, the National Institute of Environmental Research reported that lead levels are higher among children and adolescents in Korea than in the USA and Canada; mercury levels in Korea (l1.93 ug/L in the 6–11-year-old group and 1.90 ug/L in the 12–19-year-old group) were also higher than those reported in the Unites States (0.469 ug/L in 12–19-year-old individuals) and in Germany (0.23 ug/L in 6–8-year-old children and 0.22 ug/L in 9–11-year-old individuals) [75]. Further examination of the association between the incidence of proteinuria and heavy metals in drinking water is necessary.

This study has several limitations. First, as a secondary analysis, it could not be judged whether the presence of proteinuria in this study was found with the first urine in the morning, and researchers could not confirm whether the urine stick was read automatically or manually. Microalbuminuria is also a risk factor for renal disease, but the data are not available. Second, as a secondary analysis, this study did not include many different age groups. Third, this study included 16 regions because of missing regional data, so the number of regions is insufficient to completely analyze differences on the basis of regional characteristics. Additionally, this study examined data using cross-sectional research methods, which makes it difficult to determine the temporal context. Moreover, in the analysis of environmental factors related to air pollution, other elements, such as outdoor pollutants like sulfur dioxide, nitrogen dioxide, carbon monoxide, and ozone, were excluded. Further studies will need to identify whether these pollutants are also risk factors. 

## 5. Conclusions

We found significant associations between the prevalence of proteinuria and thinness in children and adolescents. To date, most previous research on the associations between BMI and abnormal urinary findings have tended to focus on overweightness and obesity. We have drawn the necessary medical attention to children and adolescents with thinness. Furthermore, we observed that environmental factors including geography, urbanization, PM_10_, heavy metals in water, and access to convenience stores affect the prevalence of proteinuria in children. In a highly industrialized and urbanized world, administrative efforts to reduce environmental health risks in urbanized or urbanizing regions and individualized intervention for managing personal factors are needed.

## Figures and Tables

**Table 1:** Proteinuria by individual characteristics (N = 27,081).

Characteristics, N (%), M ± SD	Total	Normal	Positive	*x^2^/t*	*p*
Total	27,081 (100)	26,552 (98.0)	529 (2.0)		
Sex					
Male	13,984 (51.6)	13,745 (98.3)	239 (1.7)	9.01	0.003
Female	13,097 (48.4)	12,807 (97.8)	290 (2.2)		
Grade					
First	5465 (20.2)	5433 (99.4)	32 (0.6)	116.25	<0.001
Fourth	5433 (20.1)	5358 (98.6)	75 (1.4)		
Seventh	7267 (26.8)	7041 (96.9)	226 (3.1)		
Tenth	8916 (32.9)	8720 (97.8)	196 (2.2)		
BMI ^a^					
Thinness	1802 (6.7)	1740 (96.6)	62 (3.4)	35.87	<0.001
Normal	17,751 (65.5)	17,386 (97.9)	365 (2.1)		
Overweight/obesity	7528 (27.8)	7426 (98.6)	102 (1.4)		
Systolic blood pressure	102.55 ± 12.28	102.54 ± 12.30	103.19 ± 11.29	−1.32	0.189
Diastolic blood pressure	61.69 ± 8.16	61.67 ± 8.17	62.26 ± 7.19	−1.64	0.101
Exercise					
<3 days	16,300 (60.7)	15,963 (97.9)	337 (2.1)	2.53	0.111
3 days or more	10,548 (39.3)	10,359 (98.2)	189 (1.8)		
Duration of sleep					
<8 h	18,964 (70.3)	18,537 (97.7)	427 (2.3)	28.74	<0.001
8 h or more	8005 (29.7)	7904 (98.7)	101 (1.3)		
Fast food intake					
None	7943 (29.4)	7813 (98.4)	130 (1.6)	6.04	0.014
More than once	19,029 (70.6)	18,631 (97.9)	398 (2.1)		

^a^ BMI = body mass index.

**Table 2:** Regional characteristics (N = 16).

Characteristics	M ± SD	Range	1	2	3	4	5
1. Smoking rate	22.28 ± 1.69	19.70~26.60	1				
2. PM_10_^a^	44.50 ± 4.26	38.00~53.00	−0.11 ^c^	1			
3. PM_2.5_^b^	25.13 ± 2.39	21.00~31.00	−0.18 ^c^	0.75 ^c^	1		
4. Heavy metals in drinking water	146.81 ± 168.27	0~626	0.25 ^c^	0.03 ^c^	0.07 ^c^	1	
5. The number of convenience stores per km^2^	2.12 ± 2.92	0.34~12.47	−0.46 ^c^	0.27 ^c^	0.09 ^c^	−0.19 ^c^	1

^a^ PM_2.5_ = ambient fine particulate matter with an aerodynamic diameter ≤2.5 μm; ^b^ PM_10_ = ambient air particulate matter with an aerodynamic diameter ≤10 μm; ^c^ *p* < 0.001.

**Table 3:** Proteinuria by characteristics of region (N = 27,081).

Characteristics, N (%), M ± SD	Total	Normal	Positive	*x^2^/t*	*p*
Middle-South					
Middle	14,491 (53.5)	14,246 (98.3)	245 (1.7)	11.23	0.001
South	12,590 (46.5)	12,306 (97.7)	284 (2.3)		
East-West					
East	8913 (32.9)	8682 (97.4)	231 (2.6)	28.26	<0.001
West	18,168 (67.1)	17,870 (98.4)	298 (1.6)		
Urbanization					
Metropolitan	8997 (33.2)	8793 (97.7)	204 (2.3)	19.73	<0.001
Small-medium sized city	9671 (35.7)	9464 (97.9)	207 (2.1)		
Rural area	8413 (31.1)	8295 (98.6)	118 (1.4)		
Smoking rate	22.12 ± 1.33	22.12 ± 1.33	22.07 ± 1.26	0.78	0.438
PM_10_^a^	46.10 ± 4.83	46.10 ± 4.83	46.00 ± 4.53	0.52	0.600
PM_2.5_^b^	25.83 ± 2.18	25.83 ± 2.18	25.94 ± 2.02	−1.26	0.209
Heavy metals in drinking water	172.24 ± 158.77	172.04 ± 158.41	182.26 ± 175.59	−1.33	0.185
The number of convenience stores per km^2^	2.81 ± 3.63	2.80 ± 3.62	3.21 ± 3.93	−2.39	0.017

^a^ PM_2.5_ = ambient fine particulate matter with an aerodynamic diameter ≤2.5 μm; ^b^ PM_10_ = ambient air particulate matter with an aerodynamic diameter ≤10 μm.

**Table 4:** Adjusted odd ratios (95% confidence interval) of the two-level logistic regression model for proteinuria among children and adolescents.

Variables	Null Model	Model 1	Model 2
*Fixed effect*			
**Individual level**			
Sex (ref: male)			
Female		1.25 ^f^ (1.05–1.49)	1.24 ^f^ (1.04–1.48)
Grade (ref: 1)			
4		2.52 ^h^ (1.65–3.86)	2.53 ^h^ (1.65–3.87)
7		5.75 ^h^ (3.84–8.63)	5.75 ^h^ (3.84–8.63)
10		3.88 ^h^ (2.55–5.92)	3.79 ^h^ (2.48–5.77)
BMI ^a^ (ref: normal)			
Thinness		1.76 ^h^ (1.33–2.32)	1.77 ^h^ (1.34–2.33)
Overweight/obesity		0.64 ^h^ (0.51–0.80)	0.64 ^h^ (0.51–0.80)
Duration of sleep (ref: <8hr)			
8 h or more		0.97 (0.75–1.26)	0.97 (0.75–1.26)
Fast food intake (ref: none)			
More once		1.15 (0.94–1.41)	1.15 (0.94–1.41)
**Regional level**			
Middle-South (ref: middle)			
South			2.03 ^h^ (1.39–2.98)
East-West (ref: west)			
East			1.70 ^h^ (1.30–2.24)
Urbanization (ref: Rural area)			
Metropolitan			1.48 ^f^ (1.08–2.02)
Small-medium sized city			1.51 ^g^ (1.19–1.92)
Smoking rate			1.10 ^f^ (1.01–1.20)
PM_10_^b^			1.06 ^f^ (1.01–1.11)
PM_2.5_^c^			1.01 (0.94–1.09)
Heavy metal in drinking water			1.001 ^f^ (1.0001–1.0015)
The number of convenience stores per km^2^			1.09 ^h^ (1.05–1.13)
*Random effects*			
LR ^d^ test vs. logistic regression			
Log likelihood	–2580.57	–2483.34	–2464.22
X² (*p*-value)	50.33 (<0.001)	52.44 (<0.001)	0.00 (.)
Variance (SE ^e^)			
Regional level	0.18 (0.08)	0.20 (0.09)	7.05 × 10^−33^ (1.84 × 10^−17^)

^a^ BMI = body mass index; ^b^ PM_2.5_ = ambient fine particulate matter with an aerodynamic diameter ≤2.5 μm; ^c^ PM_10_ = ambient air particulate matter with an aerodynamic diameter ≤10 μm; ^d^ LR = likelihood ratio; ^e^ SE = standard error; ^f^ *p* < 0.05; ^g^ *p* < 0.01; ^h^ *p* < 0.001.

## Data Availability

The datasets used and/or analyzed during the current study are available from the corresponding author on reasonable request.
